# Potential Neuroprotective Role of Sugammadex: A Clinical Study on Cognitive Function Assessment in an Enhanced Recovery After Cardiac Surgery Approach and an Experimental Study

**DOI:** 10.3389/fncel.2022.789796

**Published:** 2022-02-21

**Authors:** Vicente Muedra, Vicent Rodilla, Marta Llansola, Ana Agustí, Clara Pla, Antolín Canto, Vicente Hernández-Rabaza

**Affiliations:** ^1^Department of Medicine and Surgery, Faculty of Health Sciences, Institute of Biomedical Sciences, CEU Cardenal Herrera University, CEU Universities, Valencia, Spain; ^2^Department of Anesthesiology, Intensive Care Unit and Pain Therapy, University La Ribera Hospital, Valencia, Spain; ^3^Department of Pharmacy, Faculty of Health Sciences, Institute of Biomedical Sciences, Cardenal Herrera CEU University, CEU Universities, Valencia, Spain; ^4^Neurobiology Laboratory, Centro de Investigación Príncipe Felipe (CIPF), Valencia, Spain; ^5^Nutrition and Health Research Unit, Department of Microbial Ecology, Institute of Agrochemistry and Food Technology, Spanish Council for Scientific Research (IATA-CSIC), Valencia, Spain; ^6^Department of Biomedical Sciences, Faculty of Health Sciences, Institute of Biomedical Sciences, Cardenal Herrera CEU University, CEU Universities, Valencia, Spain

**Keywords:** sugammadex, postoperative cognition dysfunction, microglia, neuroinflammation, enhanced recovery after cardiac surgery

## Abstract

**Background:**

Postoperative cognitive dysfunction affects the quality of recovery, particularly affecting the elderly, and poses a burden on the health system. We hypothesize that the use of sugammadex (SG) could optimize the quality of postoperative cognitive function and overall recovery through a neuroprotective effect.

**Methods:**

A pilot observational study on patients undergoing cardiac surgery with enhanced recovery after cardiac surgery (ERACS) approach, was designed to compare SG-treated (*n* = 14) vs. neostigmine (NG)-treated (*n* = 7) patients. The Postoperative Quality Recovery Scale (PQRS) was used at different times to evaluate cognitive function and overall recovery of the patients. An online survey among anesthesiologists on SG use was also performed. Additionally, an animal model study was designed to explore the effects of SG on the hippocampus.

**Results:**

Sugammadex (SG) was associated with favorable postoperative recovery in cognitive domains particularly 30 days after surgery in patients undergoing aortic valve replacement by cardiopulmonary bypass and the ERACS approach; however, it failed to demonstrate a short-term decrease in length of intensive care unit (ICU) and hospital stay. The survey information indicated a positive appreciation of SG recovery properties. SG reverts postoperative memory deficit and induces the expression of anti-inflammatory microglial markers.

**Conclusion:**

The results show a postoperative cognitive improvement by SG treatment in patients undergoing aortic valve replacement procedure by the ERACS approach. Additionally, experimental data from an animal model of mild surgery confirm the cognitive effect of SG and suggest a potential effect over glia cells as an underlying mechanism.

## Introduction

Over the past decades, there has been a strong perception that many elderly patients undergoing surgery experience an accelerated cognitive decline beyond what is expected for normal aging, which is directly attributable to surgery and general anesthesia (Nadelson et al., 2014; Schulte et al., 2018). Certain surgeries most notably cardiac procedures, and major orthopedic surgeries have been associated with postoperative cognitive decline (POCD), which affects learning, memory, information processing, and cognitive function (Schulte et al., 2018). It has been reported that at hospital discharge, between 10 and 65% of elderly patients (>60 years of age) may be affected by POCD (Grape et al., 2012; Nadelson et al., 2014; Rundshagen, 2014; Batistaki et al., 2017; Ghaffary et al., 2017; Nemeth et al., 2017; Schulte et al., 2018).

Any persistent degree of cognitive impairment or POCD would be of concern, but there have been additional suggestions that surgery and anesthesia, particularly in elderly patients, could accelerate the onset of cognitive decline or even cause dementia (Needham et al., 2017). Cognitive dysfunction has also been associated with markedly adverse outcomes, such as a poorer functional recovery (Saczynski et al., 2012; Rundshagen, 2014), prolonged hospitalization and rehabilitation (Polunina et al., 2014; Duan et al., 2018), diminished quality of life (Saczynski et al., 2012; Brummel et al., 2014; Maniar et al., 2016), and, very commonly, work disabilities that precipitate early retirement (Steinmetz et al., 2009; Fodale et al., 2010; Leslie, 2017). Nowadays, general increase in life expectancy together with increase in scheduled cardiac surgical procedures in the geriatric population and complication such as cognitive dysfunction, could pose a significant burden on the health system (Polunina et al., 2014; Duan et al., 2018).

Currently, the quality of postoperative recovery is focused on patient-oriented endpoints and has raised an interest in the quality of anesthesia procedures (Wu and Richman, 2004; Amorim et al., 2014). In fact, prevention of cognitive impairment and rapid recovery in cognitive function are strongly encouraged in the new enhanced recovery after surgery (ERAS) program, which has shown efficacy in reducing complications and improving outcomes in many surgeries. The ERAS approach, as a multimodal and transdisciplinary care improvement initiative, aims to promote accelerated recovery of patients throughout their entire perioperative journey (Ljungqvist et al., 2017; Engelman et al., 2019) and has been recently implemented in cardiac surgery (ERACS). However, evidence-based protocols have yet to emerge (Fleming et al., 2016; Engelman et al., 2019).

Medical improvements, technical and pharmacological, probably have contributed to the decrease in postoperative mortality after cardiac surgery; however, the incidence of cognitive dysfunction has not changed, becoming a common complication (Bhamidipati et al., 2017; Glumac et al., 2019), and its prevention has not always been successful (Newman and Harrison, 2002; Royse et al., 2010; Saczynski et al., 2012). Recent studies have demonstrated that in uneventful surgery, several postoperative parameters, such as quality-of-life, patients’ functional status, and cognitive function, improve in the short/medium term (Nadelson et al., 2014; Needham et al., 2017). Other aspects, such as resolving adequately pain and/or inflammation, could be important prerequisites for postoperative cognitive improvement (POCI), which might occur even with older patients as the brain retains its neuroplastic potential throughout life (Needham et al., 2017).

Recently, some studies have pointed out that sugammadex (SG), a modified γ-cyclodextrin designed for optimal encapsulation of the neuromuscular blocking drug rocuronium (SG, StatPearls-NCBI Bookshelf) (Chandrasekhar et al., 2021), could be associated with faster recovery of consciousness after general anesthesia (Duvaldestin et al., 2010; Chazot et al., 2011; Amorim et al., 2014; Biricik et al., 2019). SG has also been shown to increase the quality of physiological recovery (Kim et al., 2019) and could reduce the incidence of POCD after general anesthesia (Batistaki et al., 2017). However, the involvement of SG in postoperative cognitive function is not fully clear. SG is a relatively novel drug, and it is, therefore, possible that the full range of its cognitive interactions has not yet been sufficiently explored.

We hypothesize that the use of SG could optimize the quality of postoperative cognitive function and overall recovery with the ERACS approach, probably through its potential neuroprotective effect. Thus, the primary endpoint of this clinical study was to compare the effect of SG with that of neostigmine (NG) on the quality of postoperative cognitive function in patients undergoing an elective aortic valve replacement procedure with the ERACS approach. Cognitive function was assessed at different time points using the Postoperative Quality Recovery Scale (PQRS). Additionally, we also aim to evaluate the effect of each treatment (SG and NG) on the quality of overall postoperative recovery and on each domain, and tried to determine whether these aspects could be correlated with the length of intensive care unit (ICU) and hospital stay.

Parallelly, an online survey was carried out to find out the opinion of anesthesiologists on postoperative recovery, particularly on cognition function, regarding the use of SG as a neuromuscular blockade (NMB) reversal agent. Likewise, an experimental study on an animal model was designed and carried out to explore the potential neuroprotective effect of SG and its mechanism of action.

## Materials and Methods

### Preliminary and Clinical Studies

#### Preliminary Study: Expert Questionnaire

In order to find out the opinion of anesthesiologists on postoperative recovery, particularly on cognitive function, regarding the use of SG, a specific online questionnaire, as a descriptive study, was prepared to take into account variables that were intended to be measured (Supplementary Appendix A). It was carried out by convenience sampling using Google Forms distributed among practicing anesthesiologists in Spain. Online access was made available between 18 December 2019 and 16 January 2020. The questionnaire consisted of 11 compulsory closed-ended questions.

#### Clinical Studies: Study Design

A single-center, observational, and prospective pilot study on patients undergoing aortic valve replacement surgery with the ERACS approach was conducted at La Ribera University Hospital (Alzira-Valencia, Spain). It was designed to evaluate the efficacy of SG compared to NG on postoperative cognitive function and recovery at different times using the Post-Operative Quality Recovery Scale (PQRS).

#### Overall Study Plan

The study was conducted in accordance with the ethical principles for medical research involving humans, as described in the Declaration of Helsinki, Convention of the Council of Europe, and Universal Declaration of UNESCO also taking into consideration the requirements of Spanish legislation in the field of research with medicines and medical devices. This research received the approval of the Hospital Ethical Committee. All the patients were fully informed about the study protocol and signed a written informed consent form.

All consecutive adult patients scheduled for elective cardiac surgery under the ERACS approach from October 2017 to February 2020 were eligible to participate in this study. In order to minimize possible biases due to different surgical procedures, the study focused exclusively on aortic valve replacement surgery with cardiopulmonary bypass (CPB), even though the ERACS program is applied to other different types of surgeries (coronary artery bypass grafting, multi-valvular, or combined procedures). Selection of patients was carried out according to defined inclusion and exclusion criteria.

Inclusion criteria were patients of either sex, at least 65 years of age, fluent in Spanish with unimpaired reading and hearing abilities, scheduled for aortic valve replacement surgery with CPB and early extubation in the operating room, and willingness to comply with all aspects of the study protocol, including the ERACS approach and PQRS assessment throughout the study period.

Exclusion criteria included non-elective surgery, pre-CPB use of intra-aortic balloon pump, ventricular assist device, and requirement for vasoactive preoperative support. Patients scheduled for coronary artery bypass grafting, multi-valvular or combined surgery (not exclusively valvular), and aortic valve repair procedures were also excluded to minimize biases that may occur because of different procedures. Patients undergoing orthopedic, neurosurgical, vascular, or previous cardiac procedures, as well as patients with neuromuscular disease and significant kidney or liver dysfunction, were also excluded. Additional exclusion criteria included (1) diagnosed psychiatric disorders, (2) any prior diagnosed disease of the central nervous system, (3) alcoholism or drug dependence, (4) medication with antidepressants and/or tranquilizers (even when these are administered 24 h before the procedure), and (5) any allergies to drugs included in the protocol.

#### Withdrawal Criteria

Patients had the right to withdraw from the study at any time for any reason (e.g., refusal to answer the PQRS). Additionally, the investigator also had the right to withdraw any patient from the study under his/her clinical judgment, which is always in the best interest of the subject (e.g., surgical failure, emergency complication, use of muscle relaxant other than rocuronium, requirement of anticholinergic drugs even after using NG, and/or extubation performed outside the operating room).

#### Study Interventions

Two groups, the SG group (*n* = 14) and the NG group (*n* = 7), were established according to the NMB reversal agent administered (SG and NG) at the discretion of the anesthesiologist in charge.

A total of 5 ml of study drugs was prepared to be administered to each patient. For patients in the NG group, a solution of 0.03 mg.kg^–1^ NG methyl sulfate (0.5 mg.ml^–1^ in a 5-ml vial; B. Braun Medical S.A; Barcelona, Spain) was prepared, whereas a solution of 2 mg.kg^–1^ of SG sodium (100 mg.ml^–1^ in a 2-ml vial; MSD Española S.A, Madrid, Spain) plus 3 ml of normal saline was prepared for patients in the SG group.

Both groups received the study drugs intravenously at the end of surgery, after confirmation of a train-of-four (TOF) ratio of 0.9 or higher (TOF 90%). Six different anesthesiologists participated in the surgical procedure but not in data collection or analysis. Two of them routinely use NG as the NMB reversal agent induced by rocuronium. The remaining participants used SG. Except for the NMB reversal agent, the rest of the anesthetic procedure was similar in both groups and adjusted to our ERACS approach.

#### Enhanced Recovery After Cardiac Surgery Protocol

Patients admitted to ERACS were previously informed about characteristics and requirements by their surgeon or anesthesiologist. A multidisciplinary team (nursing staff, physiotherapist, cardiologist, hematologist, and nutritionist) was responsible for optimizing hemoglobin levels, assessing respiratory function, implementing physiotherapeutic measures, and ensuring adequate nutrition. The program is essentially based on minimally invasive surgery (mini-sternotomy) with multimodal analgesia and short-acting anesthetic drugs to facilitate a very early extubating approach and postoperative seating and feeding re-starting within 6 h after the procedure. It should be noted that only patients extubated outside the operating room (e.g., in ICU) were excluded from this study.

#### Anesthetic Technique and Cardiopulmonary Bypass Procedure

The protocol followed for induction and maintenance of anesthesia was the same for both groups. Administration of midazolam or any other preoperative sedative drugs was strictly avoided.

Anesthesia was induced by the administration of propofol (2 to 2.5 mg.kg^–1^, intravenous, IV), fentanyl (2 mg.kg^–1^, IV), and rocuronium (0.8–1.2 mg.kg^–1^, IV). Rocuronium was administered after calibration of the accelerometry device (TOF WATCH; Organon Teknika B.V., Netherlands), which was placed on the ulnar nerve of the contralateral hand, which is opposite to the one used for intravenous drug administration. Intermittent doses of muscle relaxant (rocuronium) were used to maintain 0% TOF (TOF 0). Anesthetic maintenance was performed with a mixture of gases (02: air, 60:40) and titrating sevoflurane to achieve values of the bispectral index (BIS; Aspect Medical Systems, Newton, MA, United States) between 40 and 60. An ultrasound-guided block of the upper serratus plane was performed after anesthetic induction, and then low-dose remifentanil perfusion (0.01–0.1 mg.kg^–1^.min^–1^) was initiated. Finally, 30 min before the end of the procedure, additional paracetamol (1 g IV) completed the analgesic approach.

Together with the standard monitoring applied to any procedure performed under general anesthesia, in cardiac surgery, additional specific devices such as transesophageal echocardiography (iE33 ultrasound, Philips, Netherlands), invasive blood pressure, nasopharyngeal and rectal temperatures, and regional oxygen saturation (INVOSTM 5100C; Somanetics Corp. Troy, MI, United States) were used.

Details of anesthesia, CPB equipment, and technique, were carried as previously described (Muedra et al., 2018). Briefly, after anesthetic induction, CPB was established through a mini-sternotomy approach, aortic root cannulation, and single or bi-cava atrial cannulation for venous return. The circuit priming volume before beginning CPB was 600 ml. Antegrade intermittent cold blood cardioplegia (4:1) was used. Pump flow was set at 2.4–2.6 l.min^–1^.m^–2^, and target mean arterial pressure was set at 65–70 mmHg. Body temperature during CPB was maintained between 28 and 32°C (moderate hypothermia). All the patients received tranexamic acid intraoperatively (20 mg.kg^–1^ IV II, before the induction of anesthesia; 1 mg.kg^–1^.min^–1^ during CPB; finally, 20 mg.kg^–1^ IV after the protamine dose). Active coagulation time (ACT) was determined immediately after the induction of anesthesia, 3 min after loading heparin dose (300 IU.kg^–1^), 5 min after initiation of CPB, and subsequently every 15 min. ACT values > 460 seg or higher were considered to be satisfactory. A specific perioperative transfusion algorithm was applied to maintain hematocrit above 25%, according to clinical and hemodynamic status. Continuous insulin perfusion to maintain glycemia between 100 and 150 mg. dl^–1^ according to our hospital protocol was established. All the patients received ondansetron (4 mg IV) 30 min before the end of the procedure to prevent postoperative nausea and vomiting.

Sevoflurane and remifentanil infusion was stopped when the surgeon concluded stitching the skin. When a 25% value (TOF 25) was achieved and bispectral index score value was > 80, residual NMB was reversed by administration of either SG (2 mg.kg^–1^ IV) or NG (0.03 mg.kg^–1^ IV) gently and slowly, without anticholinergic drugs. When a 90% value (TOF 90) was achieved, the patients were extubated inside the operating room.

Once a patient was extubated and considered clinically stable, she/he was transferred to ICU initially and, afterward, to the corresponding hospital ward. Standard patient monitorization follow-ups were performed at set intervals.

#### Data Collection and Time Points

To evaluate cognitive function and overall recovery, the PQRS was used by the nursing staff responsible for data recording. All the patients were visited 12–24 h before the operation. For each patient, the following data were recorded: age, sex, height, weight, body mass index, and past medical history such as hypertension, diabetes mellitus, pulmonary disease, and chronic kidney disease. The educational level (categorized as unschooled, elementary, primary, secondary, or higher education), employment (categorized as unemployed or retired, not working (because of health reasons), or employed); alcohol consumption, smoking habits, American Society of Anesthesiologists (ASA) classification, comorbidities, and risk stratification according to EuroScore II of the patients were recorded. Similarly, for each patient, intra-operative details (e.g., CPB and aortic cross-clamp times, transfusion requirements, and vasoactive drug support) were also recorded. The length of ICU and hospital stay, as well as outcomes at discharge, were also documented.

The quality of recovery was assessed using the PQRS Spanish version, which had been translated from previous publications (Royse et al., 2010). The PQRS consisted of 6 domains of postoperative recovery (physiologic, nociceptive, emotive, cognitive, activities of daily living, and overall patient perspective). The patients were assessed at baseline using PQRS 12–24 h before surgery (T0) and then 30 min after extubation (T1), 24 h (T2), and 72 h (T3) after surgery, and finally 30 days after discharge (T4). The PQRS questionnaire was filled during a face-to-face interview with each patient in the hospital or by phone after being discharged. The patients were offered a paper copy of the PQRS to facilitate telephone assessment. Recovery was defined as the return to (or improvement from) baseline values (Royse et al., 2010). For each patient, values obtained at each time point were compared with baseline values as either recovered (return to baseline values or better) or not recovered. This was recorded for all the test items and then grouped by domain or by all domains (overall recovery). Any failure to recover for any questions within a domain rendered the whole domain as “not recovered.”

The physiological domain was assessed only during the first 72 h. The response rate of a patient from an overall perspective was assessed only 30 days post-surgery.

#### Statistical Analysis

Data were collected in a Microsoft Excel table and were analyzed using the statistical calculation program R-UCA. Statistical significance was always set at α = 0.05. Categorical data were expressed in frequencies and percentages. For continuous variables, means and standard deviation (SD) or median, range (minimum or maximum), and interquartile range (IQR, 25th-75th percentile), according to the distribution of data were calculated. For qualitative variables such as sex, employment, studies, surgery type, and items of the PQRS questionnaire, contingency tables and statistical analysis by Chi-square test were used and performed. Normality for quantitative variables, such as age, height, weight, and body mass index, was checked by the Shapiro-Wilk test, and homogeneity of variances was assessed by means of Levene test. The significance study was performed by Student’s *t*-test if the variable data were normally distributed and homoscedastic, whereas the Mann-Whitney or the Wilcoxon test (for paired data) was performed in any other cases.

### Experimental Study

#### Animal Model

Wistar rats (Charles River) housed in the animal facility of the research center Principe Felipe (CIPF; Valencia, Spain) were used for the experiments. The animals were kept in a controlled environment (12-h light/12-h darkness cycles, 23 ± 1°C, and humidity of 55 ± 5%) with access to food and water *ad libitum*. Experimental designs were approved by the Committee of Experimentation and Animal Welfare at CIPF and performed in accordance with the guidelines of the Directive of the European Commission (code: 2018/VSC/PEA/0081) for care and management of experimental animals.

#### Experimental Design

The rats were habituated for 3 weeks to the housing facilities and the researchers, and were randomly divided into four experimental groups: surgery and no-surgery and treated with SG or saline, respectively (*N* = 28, *n* = 7; named SAL and SG, for the saline and SG groups without surgery, and S-SAL and S-SG for the saline and SG with surgery groups). However, before surgery or treatment application, the rats were trained for 3 days to learn the Morris water maze task. On the fourth day, the surgery and treatments were applied. To evaluate the effect of SG on post-surgery motor recovery, once the surgery was finished, motor activity was monitored in an open field chamber, but only for the two surgery groups. One hour after the surgery, and after the motor activity assessment, the cognitive level post-surgery was analyzed in the Morris water maze. Finally, once the last test was carried out, the rats were sacrificed, and the hippocampus was dissected for further molecular and histological assessments (Figure 6A).

#### Surgery Protocol

Morphine 2% (2.5 mg.kg^–1^) was administered as preoperative analgesia. The animals were anesthetized with sevoflurane and were kept under these conditions during surgery intervention (15 min). Laparotomy was performed by making an incision up and down the abdomen (about 4 cm) with a scalpel being careful not to cut the muscle. The skin was separated from the muscle tissue with scissors. The abdominal muscle was stretched with tweezers, and a small cut was made with the scissors vertically to make a hole to cut the abdomen from bottom to top trying not to tear the muscle. After 15 min, the abdomen was sutured with absorbable silk suture 4/0 and continuous individual points with 3 knots (double, simple, and simple). The scar was cleaned and disinfected with iodine. After the sevoflurane administration ceased, SG (75 mg.kg^–1^) or the saline solution (0.9% NaCl) was injected (one dose, IP). After surgery, if typical pain behavior was observed, an animal was treated with analgesics and removed from the study.

#### Post-surgical Motor Activity Analysis

Following surgery, the rats from the surgery groups were placed in an open field activity chamber (43 cm × 43 cm × 30.5 cm) for 60 min to evaluate the effect of SG on post-surgery motor recovery. The activity was detected by arrays of infrared motion detection, with two arrays 1 cm above the floor of the chamber and another array 6 cm above the floor. The chambers were controlled via the “Activity Monitor” program (Med Associates, Pennsylvania, United States), which records motor activity, every 5 min. The apparatus recorded one ambulatory count when the rats interrupted three consecutive infrared detectors.

#### Spatial Learning in the Morris Water Maze

A behavior task designed to evaluate spatial learning and memory in rodents was used based on the Morris water maze test (Morris, 1984), which uses a circular water pool (160 cm diameter, 40 cm height) arbitrarily divided into four quadrants. After habituation to the pool for 90 s, the rats were trained to learn and locate an invisible platform for 3 days before surgery (called training period). Each training trial involved placing a rat into the pool facing the wall of one of the three quadrants that did not contain the submerged platform. In each trial, a different starting point was randomly used. The training consisted of 3 trials per day. Each animal was allowed a maximum of 120 s to find the platform and was left for 20 s on the platform. If a rat failed to locate the platform within 120 s, it was then manually guided to the platform by the experimenter. Twenty-four hours after finishing the training period, surgery and treatments were applied. After surgery and the following assessment of motor activity, all the experimental groups were subjected to one final trial in the Morris water maze. In all the Morris Water maze sessions, the time needed and the average swimming speed to find the hidden platform were recorded with and video camera and specific software (ViewPoint Behavior Technology, France). To analyze cognitive recovery, a learning index was used. The index was the result of time spent in the quadrant containing the platform on day 4 (D4, post-surgery) minus the same time during the third trial of day 3 (D3 pre-surgery; baseline values).

During the water maze training sessions, five rats (two in each of the surgery groups and one in the control saline group) displayed stress signals, affecting the exploration and task learning, and were excluded from the experiment.

#### Analysis of Protein Content by Western Blotting

Homogenates of the hippocampus were subjected to immunoblotting as previously described (Felipo et al., 1988). The samples were codified and sent to the laboratory of neurobiology at the center Príncipe Felipe (Valencia, Spain) for analysis. Primary antibodies against Interleukin 4 (IL-4), ionized calcium-binding adaptor molecule 1 (Iba1), chitinase 3-like 3 antibody (YM1) (1:2,000), differentiation cluster 68 (CD68) (1:500), purinergic ionotropic P2 × 7 receptor (1:8,000) were obtained from Abcam (Cambridge, United Kingdom), and glial fibrillary acidic protein (GFAP) (1:5,000) was obtained from Sigma (St. Louis, MO, United States). As a control for protein loading, the membranes were incubated with anti-actin (Abcam, Cambridge, MA United States; 1:1,000). Secondary antibodies (1:2,000) were conjugated with alkaline phosphatase (Sigma, St. Louis, MO, United States). Images were captured using ScanJet 5300C (Hewlett-Packard, Amsterdam, Netherlands), and band intensities were quantified using Alpha Imager 2200 version 3.1.2 (Alpha Innotech Corporation, San Francisco, CA, United States).

#### Microglia: Immunohistochemistry Analysis

Three rats per group were anesthetized with sodium pentobarbital and transcardially perfused with 0.9% saline followed by 4% paraformaldehyde in.a 1-M phosphate buffer (pH 7.4). Brains were postfixed in the same fixative solution for 24 h at 4°C. Tissues were processed for paraffin embedding on a Leica ASP300 tissue processor (Leica Microsystems, Buffalo Grove, IL, United States). Five-micrometer-thick paraffin-embedded horizontal sections were cut and mounted on a coated slide glass. Tissue sections were then processed with the Envision Flex + Kit (Agilent Technologies, Santa Clara, CA, United States) and incubated with the primary antibody [anti–ionized calcium-binding adapter molecule 1 (Iba1; 019-19741; Wako Pure Chemicals, Tokyo, Japan) or the GFAP from Sigma (St. Louis, MO, United States). The reaction was visualized with Envision Flex + horseradish peroxidase and diaminobenzidine. The sections were counterstained with Mayer’s hematoxylin (S3309; Agilent Technologies, Santa Clara, CA, United States). The stained slices were scanned with a Pannoramic Scan slice scanner (3DHistech, Budapest, Hungary) to obtain a whole image of a slice. Once scanned, amplified pictures of the regions of interest (at 63x magnification) were recorded with the Pannoramic viewer software. Immunohistochemical quantification was performed using ImageJ v. 1.48 (National Institutes of Health, Bethesda, MD, United States). Three images from the hippocampus were taken from each slice, the dentate gyrus, molecular layer, and CA1 region using three slices per animal. To analyze microglial (Iba1) or astrocyte (GFAP) expressions in the hippocampus, the images were digitized and converted to gray scale, and the positive area was quantified.

To quantify number and length branches, immunohistochemical photomicrographs were converted into skeletonized images and analyzed using the ImageJ software plugins Analyze Skeleton and FracLac according to a method described (Young and Morrison, 2018).

#### Statistical Analysis

The results are presented as the mean ± standard error of the mean (SEM). Statistical significance was evaluated by one-way ANOVA followed by Tukey’s multiple comparisons test. *P* values < 0.05 were considered statistically significant. Statistical analysis was performed using the Graph Pad Prism 4 software (GraphPad Software Inc. San Diego, CA, United States).

## Results

### Preliminary Study: Expert Questionnaire

The questionnaire was answered by 180 practicing anesthesiologists, of whom 54% had more than 16 years of experience, whereas only 7% had less than 5 years of experience. Most responding anesthetists (31.7%) performed their duties in general surgery, 25.6% of them were associated with traumatology, 16.1% with cardiothoracic surgery, 5.6% with otorhinolaryngology, and 21.1% with other areas.

For rapid sequence intubation, 64.7% of those surveyed used rocuronium as a muscle relaxant and 31.7% used suxamethonium. Neuromuscular transmission monitoring (TOF) was used by 38.8% of the respondents. Rocuronium, a neuromuscular blocker agent, was used during the induction and maintenance phases of general anesthesia by 77.2% of those who answered the questionnaire.

**FIGURE 1:**
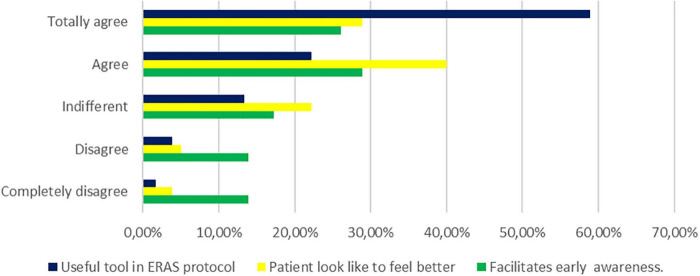
Perception of the use of sugammadex in daily clinical practice by surveyed anesthesiologists. The degree of conformity is evaluated by three essential questions: (dark blue group) useful tool in enhanced recovery after surgery (ERAS) protocols, (yellow group) patient looks like feeling better after surgical procedures, and (green group) sugammadex could facilitate early awareness.

The results of the analysis of issues of concern (question numbers 7, 8, and 9) are summarized in Figure 1. Over 55% of the respondents were in agreement (answered “agree or totally agree”) to the fact that SG accelerated the recovery of consciousness/awareness regardless of its role in reversing muscle function (question 7); 68.9% of the anesthesiologists also agreed that SG optimized the subjective feeling and well-being of patients after the anesthetic procedure (question 8). Finally, more than 80% of the anesthetists were in agreement (“agree or totally agree”) that SG was an essential tool in the ERAS program (question 9).

Sugammadex (SG) was easily available at their workplace for 77.2% of the anesthesiologists. Among the most frequently observed side effects, 4.4% reported nausea/vomiting, 11.7% bronchospasm, and 16.1% reported other effects such as dysgeusia, laryngospasm, trismus, weakness, muscle stiffness, and urticaria/anaphylaxis. Most anesthetists (67.8%), however, did not report any adverse effects.

The complete English version of the questionnaire is shown in Supplementary Appendix A.

### Clinical Study

**FIGURE 2:**
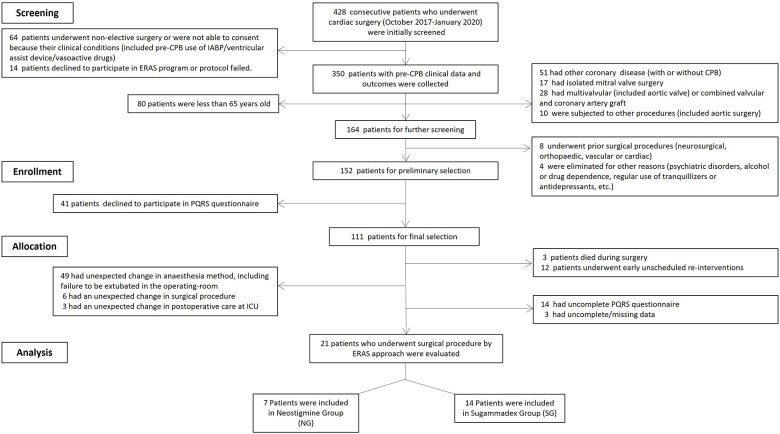
Flow chart showing the patient selection process. Initially, a total of 428 cardiac surgery patients were recruited, but 407 did not meet the inclusion criteria; finally, 21 patients were included in the study, seven of whom (33.3%) were included in the neostigmine cohort group (NG), and 14 (66.7%) in the sugammadex cohort group (SG).

This pilot study was conducted from October 2017 to January 2020. Of the 428 patients recruited, 276 did not meet the inclusion criteria. Of 152 patients, 41 declined to answer the PQRS questionnaire. A further 58 patients already allocated to a group were withdrawn because of unexpected/ongoing adjustments in surgery, anesthesia, and/or postoperative care management, and a further 12 were admitted for early re-operation and excluded. Seventeen patients did not complete the PQRS questionnaire or had to be excluded from analysis because of incomplete or missing data. A further three patients died during surgery or on the first postoperative day. Finally, of the 21 patients included in the study, 66.7% (*n* = 14) were included in the SG and 33.3% (*n* = 7) in the NG groups. The screening, eligibility, and enrollment of patients are shown in Figure 2.

Demographic characteristics and anesthesia and surgery-related data are shown in Table 1. Patients in the SG group were older than those in the NG group, although differences were not statistically significant. Although participants allocated to the SG group had higher level of education and higher risk of mortality (EuroScore II), the differences were not statically significant. The length of hospital stay for patients in the NG group was significantly shorter than that for those in the SG group (3.1 ± 0.9 vs. 4.2 ± 1.2; *p* = 0.049).

**TABLE 1:** Baseline characteristics of patients with anesthesia and surgery-related data.

	NG	SG	*p*-value
Patients Age (years old)	*n* = 7 70 ± 5	*n* = 14 74 ± 3	0.082
BMI (kg/m^2^)	29.1 ± 4.5	27.8 ± 5.8	0.607
Weight (kg)	78 ± 14.5	76.1 ± 13.6	0.827
Size (cm)	163 ± 9.9	161.9 ± 6	0.761
Male, gender, *n* (%)	4 (57.1)	11 (78.6)	0.306
Education			
No education, *n* (%)	0	0	0.135
Elementary, *n* (%)	1 (14.3)	8 (57.1)	
Primary, *n* (%)	5 (71.4)	3 (21.4)	
Secondary, *n* (%)	1 (14,3)	2 (14.3)	
Higher education, *n* (%)	0	1 (7.1)	
Employment status			
Retired, *n* (%)	6 (85.7)	14 (100)	0.147
Not-working, *n* (%)	1 (14.3)	0	
Active, *n* (%)	0	0	
EuroScore II	2.15 ± 0.7	3.18 ± 1.33	0.071
Time to anesthesia (min)	247 ± 46.9	273 ± 47.6	0.243
Surgery time (min)	210.7 ± 48.2	223.6 ± 56	0.613
Ischemia time (min)	62.3 ± 23.4	59.7 ± 17.3	0.799
CPB time (min)	91 ± 34.7	77 ± 18	0.378
Length of ICU stay (days)	1.8 ± 0.7	3.4 ± 3.2	0.376
Length of hospital stay (days) Total stay (days).	3.14 ± 0.9 5 ± 1.2	4.2 ± 1.2 7.5 ± 3.8	0.049* 0.053

Data from categorical variables are expressed in frequencies and percentages; data from continuous variables are means ± standard deviation. BMI, body mass index; min, minutes; CPB, extracorporeal circulation; EuroScore II, European system for cardiac operative risk evaluation (logistic); ICU, intensive care unit; SG, sugammadex group; NG, neostigmine group. Statistical significance was always set at a = 0.05; *p < 0.05.

Concerning the physiological domain, the percentage of patients “fully awake (consciousness)” and, similarly, in the “obeying verbal commands” category (e.g., lift your head, touch your nose, etc.), at 30 min, was 85.7% in the SG group and 42.9% in the NG group (*p* = 0.04) (Table 2).

**TABLE 2:** Physiological domain in the Postoperative Quality Recovery Scale (PQRS).

Physiological domain	NG	SG	*p*-*value*
**T1 (30 min)**			
Systolic blood pressure	85.7	78.6	0.694
Heart rate	100	92.9	0.468
Temperature	57.1	85.7	0.147
Respiration	100	92.9	0.468
Oxygen	57.1	71.4	0.512
Airway control	100	78.6	0.185
Agitation	100	92.9	0.468
Consciousness (fully awake)	42.9	85.7	0.04*
Obeying verbal commands	42.9	85.7	0.04*
**T2 (24 h)**			
Blood pressure	85.7	100	0.147
Heart rate	100	100	0.126
Temperature	85.7	100	0.147
Respiration	100	85.7	0.293
Oxygen	100	85.7	0.293
Airway control	100	85.7	0.293
Agitation	100	92.9	0.468
Consciousness (fully awake)	100	100	0.126
Obeying verbal commands	100	100	0.126
**T3 (72 h)**			
Blood pressure	71.4	92.9	0.185
Heart rate	100	100	0.126
Temperature	85.7	100	0.147
Respiration	100	85.7	0.293
Oxygen	100	85.7	0.293
Airway control	100	85.7	0.293
Agitation	100	100	0.126
Consciousness (fully awake)	100	100	0.126
Obeying verbal commands	100	100	0.126

Recovery rates compared to baseline (T0) expressed as percentage of patients for the different items in the physiological domain. Data were obtained at different times of the study: T1 = 30 min after surgical procedure, T2 = 24 h, and T3 = 72 h. Sugammadex group (SG). Neostigmine group (NG). Statistical significance was always set at a = 0.05; *p < 0.05.

No statistically significant differences among the groups were identified in each category of all the domains, except for the nociceptive (patients showed better pain control) and cognitive domains at the T4 time point (Table 3). Our findings demonstrated more favorable recovery (relative to the percentage of patients) in the SG group than in the NG group for the nociceptive (100 vs. 71.4%, *p* = 0.035) and cognitive domains (85.7 vs. 42.9%, *p* = 0.04) at the T4 time point (Table 3).

**TABLE 3:** Overall recovery rates and recovery rates by domains in the PQRS at different time points.

	NG	SG	*p*-*value*
**T1 (30 min)**			
Overall recovery	0	0	0.126
Physiological	1/7 (14.3%)	6/14 (42.9%)	0.190
Emotional	6/7 (85.7%)	13/14 (92.9%)	0.599
Nociceptive	1/7 (14.3%)	1/14 (7.1%)	0.599
Cognitive	0	0	0.126
Daily life activities	0	0	0.126
**T2 (24 h)**			
Overall recovery	0	0	0.126
Physiological	3/7 (42.9%)	9/14 (64.3%)	0.349
Emotional	4/7 (57.1%)	11/14 (78.6%)	0.305
Nociceptive	3/7 (42.9%)	4/14 (35.7%)	0.750
Cognitive	3/7 (42.9%)	3/14 (21.4%)	0.305
Daily life activities	0	1/14 (7.1%)	0.468
**T3 (72 h)**			
Overall recovery	1/7 (14.3%)	2/11 (14.3)	1
Physiological	4/7 (57.1%)	12/14 (85.7%)	0.147
Emotional	6/7 (85.7%)	9/14 (64.3%)	0.305
Nociceptive	6/7 (85.7%)	11/14 (78.6%)	0.694
Cognitive	3/7 (42.95%)	6/14 (42.9%)	1
Daily life activities	3/7 (42.9%)	4/14 (28.6%)	0.512
**T4 (30 d)**			
Overall recovery	3/7 (42.9%)	10/14 (71.4%)	0.203
Emotional	7/7 (100%)	13/14 (92.9%)	0.468
Nociceptive	5/7 (71.4%)	14/14 (100%)	0.035*
Cognitive	3/7 (42.9%)	12/14 (85.7%)	0.040*
Daily life activities	7/7 (100%)	13/14 (92.9%)	0.468

Data expressed as recovered patients/total patients (%, percentage). SG, sugammadex group; NG, neostigmine group. *p < 0.05.

The percentage of patients who remembered to answer “their name correctly, date of birth, and place in which she/he is” was significantly higher in the SG group (92.9 vs. 57.1%; *p* = 0.049) 30 min after surgery (T1) (Table 4). Additionally, among the several categories within the cognitive domain, a large majority of patients in the SG group were able to remember more words from a previously read list (“word list” category) than those who recalled in the NG group (92.9 vs. 42.9%, *p* = 0.011) at the T3 time point. Similarly, they were able to come up with more words beginning with the letter “F” (“word generation” category) (78.6 vs. 28.6%, *p* = 0.026) at the same time point. This statistically significant difference was also present 30 days after surgery (T4), at least for the “word list” category (92.9 vs. 57.1%, *p* = 0.049), and probably it contributed to improving the “global” category (85.7 vs. 42.9%, *p* = 0.04), at this time point (T4).

**TABLE 4:** Percentages of patients in the different items of the cognitive domain at different time points.

Cognitive domain	NG	SG	*p*-value
**T1 (30 min)**			
Global	0	0	0.127
Name, date, place	4/7 (57.1%)	13/14 (92.9%)	0.049*
Digits forward	0/7 (0%)	3/14 (21.4%)	0.186
Digits backward	1/7 (14.3%)	2/14 (14.3%)	1.0
Word list	0/7 (0%)	4/14 (28.6%)	0.116
Word generation (letter ‘F’)	2/7 (28.6%)	4/14 (28.6%)	1.0
**T2 (24 h)**			
Global	3/7 (42.9%)	3/14 (21.4%)	0.305
Name, date, place	7/7 (100%)	13/14 (92.9%)	0.469
Digits forward	5/7 (71.4%)	9/14 (64.3%)	0.743
Digits backward	5/7 (71.4%)	7/14 (50.0%)	0.350
Word list	4/7 (57.1%)	11/14 (78.6%)	0.305
Word generation (letter ‘F’)	2/7 (28.6%)	8/14 (57.1%)	0.217
**T3 (72 h)**			
Global	3/7 (42.9%)	6/14 (42.9%)	1
Name, date, place	7/7 (100%)	14/14 (100%)	0.127
Digits forward	5/7 (71.4%)	8/14 (57.1%)	0.525
Digits backward	5/7 (71.4%)	9/14 (64.3%)	0.743
Word list	3/7 (42.9%)	13/14 (92.9%)	0.011*
Word generation (letter ‘F’)	2/7 (28.6%)	11/14 (78.6)	0.026*
**T4 (30 d)**			
Global	3/7 (42.9%)	12/14 (85.7%)	0.040*
Name, date, place	7/7 (100%)	14/14 (100%)	0.127
Digits forward	6/7 (85.7%)	13/14 (92.9%)	0.599
Digits bakward	4/7 (57.1%)	12/14 (85.7%)	0.147
Word list	4/7 (57.1%)	13/14 (92.9%)	0.049*
Word generation (letter ‘F’)	6/7 (85.7%)	14/14 (100%)	0.147

Data expressed as recovered patients/total patients (%, percentage). NG, neostigmine group; SG, sugammadex group. *p < 0.05.

**FIGURE 3:**
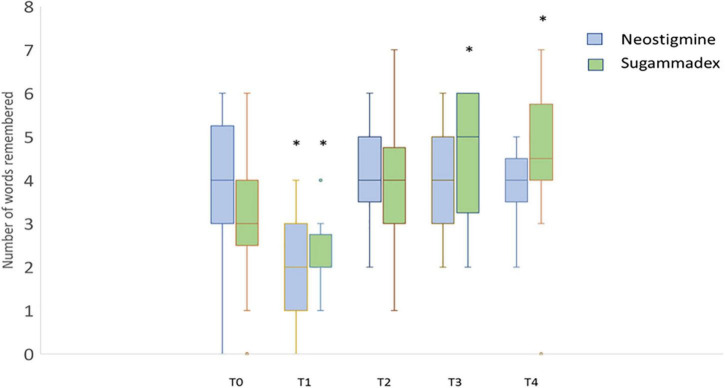
Cognitive domain: word list category. Boxplot diagram showing the effects on cognitive level by asking a patient to repeat as many words as he/she could remember from a list, at different times before surgery (T0) and 30 min (T1), 24 h (T2), 72 h (T3), and 30 days (T4) after surgery. Statistically significant differences (*p < 0.05) were detected at 30 min both for neostigmine (*p* = 0.017) and sugammadex (*p* = 0.008), and 72 h (*p* = 0.037) and 30 days (*p* = 0.043) after surgery in patients receiving sugammadex when compared to data obtained prior to surgery (T0). Neostigmine-treated patients (blue) and sugammadex-treated patients (green).

**FIGURE 4:**
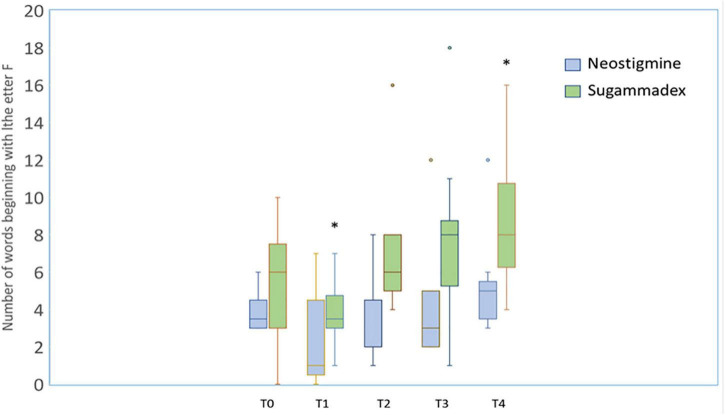
Boxplot diagram showing the effects of neostigmine (blue) and sugammadex (green) on cognitive level by asking a patient to name as many words as he/she could beginning with the letter “F” at different times before surgery (*t* = 0, T0) and 30 min (T1), 24 h (T2), 72 h (T3), and 30 days after surgery (T4). No differences were found among different times in patients receiving neostigmine, whereas statistically significant differences (*p < 0.05) were detected 30 min (T1) (*p* = 0.007) and 30 days (T4) (*p* = 0.005) after surgery when compared to data obtained prior to surgery (T0).

The number of correct answers in the different categories of the cognitive domain was also evaluated. It should be noted that this evaluation is carried out by comparing the number of correct responses that each individual achieves at different time points (T1–T4) relative to his/her own baseline responses (T0). Thus, in the “word list” category, a significant decrease in words correctly remembered was observed for both groups at T1; however, at T3, this trend was positively reversed, registering a statistically significant increase in the number of words correctly remembered in the SG group, and this difference was also maintained at T4 (Figure 3). Similarly, in the “word generation” category in the cognitive domain, statistically significant differences were observed in the SG group at T1 and T4 (Figure 4).

In the nociceptive domain, patients in the SG group reported significantly lower pain 30 days after surgery (1.07 ± 0.13 vs. 1.28 ± 0.13, *p* = 0.015). These data were assessed with a Likert-type scale of 1 to 5 points (where higher scores indicated more pain). However, regarding nausea/sickness, no differences were observed between the two groups.

The global perspective of the patients relative to the effect of the surgery was more favorable for those treated with SG in terms of working capacity (64.3 vs. 57.1%) and daily activities (71.4 vs. 42.9%) 30 days after the surgical intervention, although the differences were not statistically significant. The degree of satisfaction with anesthetic care was considered similar in both groups: “very satisfied.”

### Experimental Study

#### Behavior Analyses: Post-surgical Motor Activation Assessment

**FIGURE 5:**
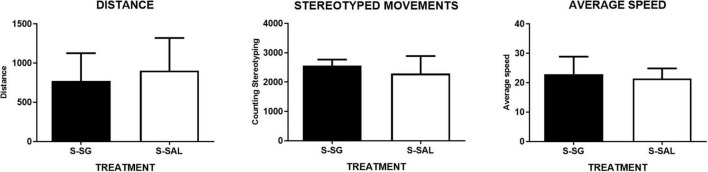
Post-surgical motor activation assessment. Motor activity graphs. No differences between both surgery groups treated with sugammadex (S-SG; *n* = 5) or saline (S-SAL; *n* = 6) were detected. The data were analyzed by Student *t*-test for distance, (**left** graph) (means ± SEM: 906.3 ± 170.4 (S-SAL); 773 ± 157.4 (S-SG); *p* = 0.595), stereotypic movements (**middle** graph) (2291 ± 243.6 (S-SAL); 2,560 ± 93.68 (S-SG); *p* = 0.366), and mean speed, (**right** graph) (means: 21.43 ± 1.398 (S-SAL), 22.8 ± 2.712 (S-SG), *p* = 0.647).

Just immediately after the surgery, while the rats were recovering from the surgery, motor activity parameters were recorded for 1 h in open chambers. This analysis was performed to evaluate the effect of SG on motor recovery after surgery. No differences between both surgery groups treated with SG (S-SG; *n* = 5) or saline (S-SAL; *n* = 6), were detected. The data were analyzed, by means of Student *t*-test, for distance (means ± SEM: 906.3 ± 170.4 (S-SAL); 773 ± 157,4 (S-SG); *p* = 0.595), stereotypic movements (2,291 ± 243.6 (S-SAL); 2560 ± 93.68 (S-SG); *p* = 0.366), and mean speed (means: 21.43 ± 1.398 (S-SAL), 22.8 ± 2.712 (S-SG), *p* = 0.647). These data are presented in Figure 5.

#### Behavior Analyses: Spatial Learning Analysis in the Morris Water Maze

**FIGURE 6:**
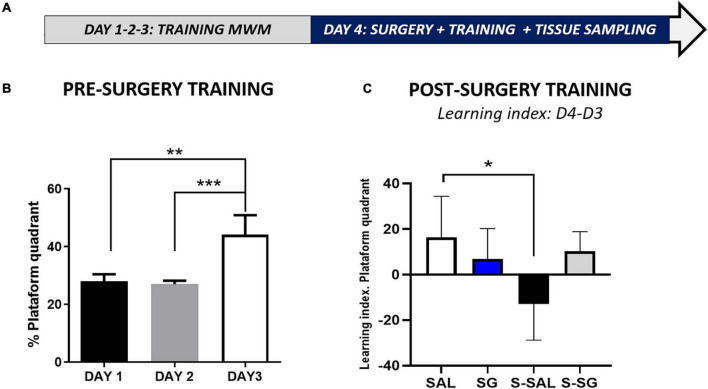
Spatial learning analysis on the Morris water maze. **(A)** Chronogram detailing the pre- and post-training periods, including the Morris mater maze training sessions, surgery where post-surgery activity recovery was included, posterior Maze training, and final tissue sampling. **(B)** Pre-surgery training in the Morris water maze, the percentage of time spent in the platform quadrant is represented, note that for the sake of clarity data for all four groups have been merged to show the learning as achieved by day 3, all the animals learning the task, with no detected animal differences. **(C)** Morris mater maze, post-training sessions and learning index: percentage of time spent in the platform difference between day 4 (D4) post-surgery and day 3 (D3) pre-surgery. SAL, saline group, no surgery; SG, Sugammadex group, no surgery; S-SAL, Surgery group treated with saline; S-SG, Surgery group treated with sugammadex. Statistical significance was evaluated by one-way ANOVA followed by Tukey’s multiple comparisons test; *p < 0.05; **p < 0.01; ***p < 0.001.

1. Pre-surgery, Morris Water Maze training. All the experimental animals learned to find the platform during the training period (3 days, 3 sessions per day). Learning was assessed by the time spent in the platform quadrant through the training period. The statistical analysis indicated a significant difference among the groups, (one-way ANOVA, *p* = 0.0004), and subsequent *post-hoc* test signaled significant differences between the first and second training days with the third (Figure 6B). Means ± SEM, day 1: 28.02 ± 2.478; day 2: 27.06 ± 1.156; day 3: 44.21 ± 6.704 (Tukey’s multiple comparisons test, day 1 vs. day 3, *p* = 0.001 ^**^; day 2 vs. day 3, *p* = 0.007). To see additional statistical data and the values for each group, see Supplementary Material.
2. Post-surgery, training sessions. After the surgery, the rats had a new training session. No differences were found concerning the average swimming speed among the groups (*p* = 0.286; more details in Supplementary Data), indicating no motor impairments by the surgery. To assess the effects of surgery on cognitive skills, a learning index was used (platform quadrant time, D4-D3). The results indicate slight cognitive impairment because of surgery that was not shown in the surgery group treated with SG, suggesting SG effect on cognitive post-surgery recovery. A one-way ANOVA indicated a significant difference among the groups (*p* = 0.024); *post-hoc* Tukey’s multiple comparisons tests revealed statistical differences between the SAL group and the S-SAL group (*p* = 0.177) (see Figure 6C).

#### Neuroinflammation Marker Analysis on the Hippocampus

Spatial learning and memory depend on hippocampus activity (Eichenbaum et al., 1990). Hippocampus inflammation impairs neural activity and reduces learning and memory. Neuroinflammation has been reported as one of the underlying mechanisms of post-surgery cognitive impairment (Lin et al., 2020). To assess the inflammation of the hippocampus and effects of SG on inflammatory response, several inflammatory markers were analyzed by Western blot and immunohistochemistry.

**FIGURE 7:**
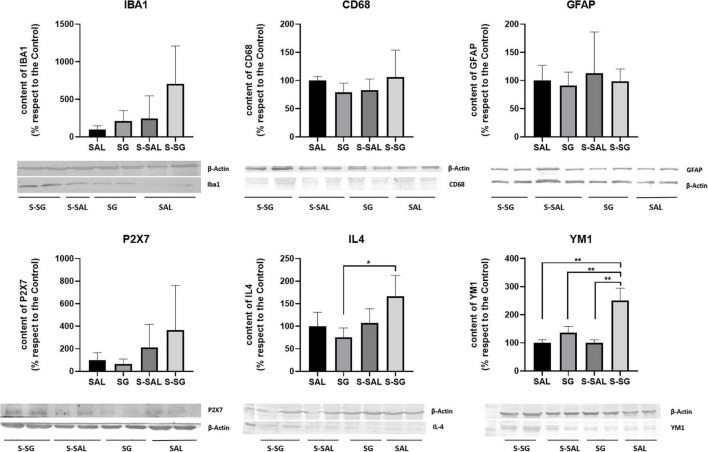
Neuroinflammation marker analysis on the hippocampus. Represented series of inflammatory markers, cellular and mediator, were analyzed by Western blot. The markers and control actin bands are shown. No significant differences were detected for the astrocyte marker GFAP (*p* = 0.899), the classical microglia Iba1 (*p* = 0.074), CD68 (*p* = 0.742), and P2 × 7 (*p* = 0.189) markers. IL4 and YM-1 are both considered M2 markers. Significant differences were found in IL4 (*p* = 0.026) and YM-1 (*p* = 0.003) assessment. Statistical significance was evaluated by one-way ANOVA followed by Tukey’s multiple comparisons test, *p < 0.05; **p < 0.01.

For initial screening and to build up a panoramic view, classical glial markers were assessed by Western blot. No significant differences were detected for the astrocyte marker GFAP (*p* = 0.899) or the classical microglia marker Iba1 (*p* = 0.074). In the second screening, we analyze inflammatory markers, such as the CD68 marker, which is a macrophage marker associated with microglia activation states, especially in damaged tissues (Hendrickx et al., 2017). No differences were detected in levels of protein CD68 expression (*p* = 0.742). To further evaluate the activation of the inflammation process, we analyzed the purinergic receptor P2 × 7, which has been proposed to act as a modulator and initiator of inflammation and microglial activation (Fabbrizio et al., 2017). The statistical analysis indicated no significant P2 × 7 differences (*p* = 0.189), but slight increase in mean values of the surgical groups, in comparison with the no surgical groups values, was found. As the final step of the initial screening, we analyzed markers of alternative microglia activation. Neuroinflammation is a dynamic process that has been linked with an alternative microglia activation state between the polarization M1-M2 states, which are inflammatory and anti-inflammatory, respectively. IL4 and YM-1 are both considered M2 markers. In the western blot analysis, significant differences were found in IL4 assessment (*p* = 0.026), with considerable increase in the S-SG group in comparison to the SAL group (Tukey’s test, *p*-value = 0.018). In the same line, the ANOVA for the YM-1 marker data also showed significant differences among the groups (*p* = 0.003), particularly between the surgical group treated with SG and the rest of the experimental groups, as was revealed by Tukey’s test (*p*-values in comparison with the group S-SG, groups: SAL (*p* < 0.0001); SG (*p* = 0.0007); S-SAL (*p* = 0.0004), and all the results are presented in Figure 7.

For a deep analysis of microglia and astrocyte activation, we analyzed the hippocampus expression of the microglia marker Iba-1 and GFAP astrocyte marker by immunohistochemistry. The results indicate a significant alteration in Iba-1 expression pattern among the groups (ANOVA, *p*-value = 0.085), and additional Tukey’s test pointed out significant differences between the SAL and S-SAL groups (*p* = 0.049) a difference that was not presented in the surgery group treated with SG. Moreover, the SG group expressed a significantly increased expression in comparison with the SAL group (*p* = 0.0064). However, no differences in GFAP expression were found in any of the three regions analyzed (GFAP results are detailed in additional Supplementary Data). For more details, see Supplementary Material.

**FIGURE 8:**
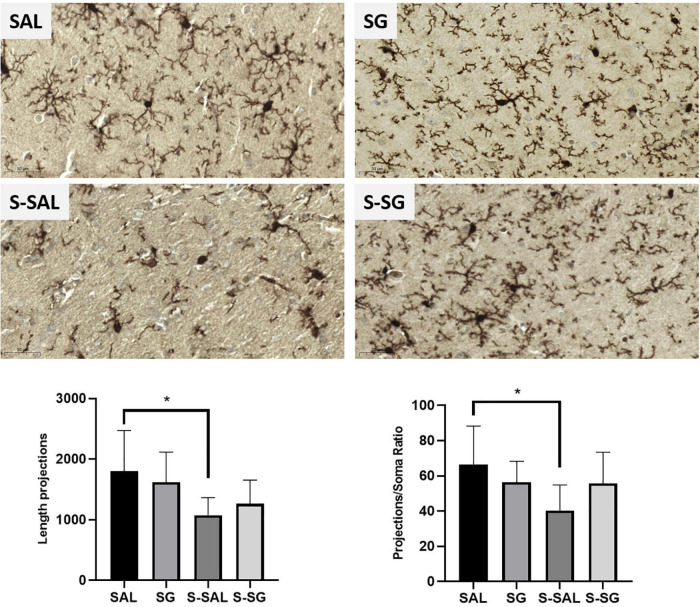
Microglia immunohistochemistry analysis. Representative images of the microglia marker Iba1, (brown mark) in the region proximal to the hippocampus molecular region. Below, graphs from the length branch assessment and branches/soma ratio results. Images and graph, SAL and SG, groups without surgery treated with saline or sugammadex, respectively, and S-SAL and S-SG, groups with surgery treated with saline or sugammadex, respectively. Statistical significance was evaluated by one-way ANOVA followed by Tukey’s multiple comparisons test, *p values < 0.05.

Finally, microglia activation is characterized by morphological changes, including the length shortening of its branches (length projections) and proportion of branch length in relation to soma diameter (projections/soma ratio). The morphological microglia analysis indicated a significant difference among the groups, including the length projections of microglia branches (*p* = 0.025) between the SAL and S-SAL groups (*p*-value = 0.033, Tukey’s multiple comparisons test). These results were similar in the assessments of branches and soma ratio (ANOVA, *p* = 0.0483;and *p* = 0.0287, Tukey’s multiple comparisons test). In summary, the results pointed out microglia activation in the group with surgery that was reduced by the SG treatment (see Figure 8).

The results presented in this last section are novel but preliminary data, which suggest a possible effect of SG on the post-surgery neuroinflammation process. Future research is required to confirm or refute the possible role of SG in neuroinflammation.

## Discussion

### Preliminary Study: Expert Questionnaire

Our aim is particularly focused on postoperative cognitive recovery with attention to the recent collective effort to build the ERACS approach. Thus, we have tried to explore the potential beneficial effects of SG as a pharmacological implementation on the quality of postoperative recovery in this scenario.

For this purpose, an online questionnaire was sent out to be answered by a sample of Spanish anesthesiologists. Over 55% of the experts surveyed were in agreement (“to be agree or totally agree”) that SG facilitated an accelerated recovery of alertness (or consciousness) after an intervention under general anesthesia. This percentage was increased by up to 70% when they answered about “the subjective feeling that the patient seems to feel better” after NMB reversal with SG. These results are consistent with previous reports (Amorim et al., 2014), revealing the potential effect of SG on the widespread perception of well-being and alertness of patients undergoing general anesthesia. These are decisive factors to optimize postoperative functional recovery and, particularly, cognitive function.

Several studies including some randomized controlled trials have shown that SG provides faster and complete reversal of NMB than NG (Khuenl-Brady et al., 2010; Geldner et al., 2012; Keating, 2016), and that it has a favorable safety profile (Naguib, 2007; Bom et al., 2009; Hristovska et al., 2018). Its direct action mechanism by encapsulating rocuronium has contributed to anesthesia protocols enabling fast and complete reversal of NMB (Bom et al., 2002; Donati, 2008). As reversal of moderate to deep NMB was attempted in most of these studies, the residual NMB was likely one of the main factors affecting the quality of postoperative recovery (Bom et al., 2009; Naguib and Brull, 2009; Bailey, 2017).

Most anesthesiologists consulted (more than 80%) agreed on the wide use and benefits of SG, thus highlighting its potential usefulness as a therapeutic tool in ERAS approaches, accelerating the recovery of consciousness and patient feeling of well-being after general anesthesia. To date, its greatest advantage has been optimization of fast and powerful reversal of NMB, even moderate to deep, induced by rocuronium. However, this reversal of NMB does not explain why patients seem to recover full alertness and consciousness faster after general anesthesia. In our opinion other, hitherto, not fully investigated characteristics of these reversal agents could also be involved. In any case, our survey did not raise the question as to whether the qualities of improvement in the postoperative recovery of patients after the use of SG could be related to a powerful and complete reversal of the NMB achieved with this drug.

The current knowledge of the effects of SG on consciousness or even cognitive recovery is rather scarce (Amorim et al., 2014; Batistaki et al., 2017; Fassoulaki et al., 2017; Paech et al., 2018), especially as it is a drug whose therapeutic indication is not linked to this aspect.

### Clinical Study

This clinical study has tried to explore the effect of SG on the improvement of overall recovery and on each PQRS domain, focusing especially on cognitive function. Our results showed that SG, for reversal of NMB and compared with NG, was associated with more favorable postoperative recovery in the nociceptive and cognitive domains, but only in the later period of our study (T4). However, as this is an observational pilot study, the results need to be interpreted cautiously.

Evaluation of cognitive function after cardiac surgery is complicated. A significant obstacle of neuropsychological assessment represents the absence of a worldwide-accepted POCD definition (Bhamidipati et al., 2017; Glumac et al., 2019), as well as different independent risk factors involved such as surgery (Bhamidipati et al., 2017; Glumac et al., 2019), individual variation in patients (Duan et al., 2018; Glumac et al., 2019), and/or anesthesia-related factors (Piskin et al., 2016; Glumac et al., 2019).

The multiple aspects of postoperative recovery, not exclusively those in the cognitive domain, allowed for a far more complex assessment of the recovery process than the currently available using other scales of recovery (Royse et al., 2010); for this reason, the PQRS as an assessment scale was chosen. Certainly, the PQRS is not formally assessing POCD but cognitive recovery, allowing for a practical and simple appraisal of it. Additionally, several authors have highlighted that delayed cognitive recovery within the first 3 postoperative days could be a strong predictor of POCD development (Glumac et al., 2019). These data are consistent with other publications associating the duration of anesthesia with recovery parameters or morbidity indicators and, therefore, add further value to the use of the PQRS (Royse et al., 2010).

This study shows that the use of SG for the reversal of NMB in patients undergoing an elective aortic valve replacement procedure following an ERACS approach optimized the quality of postoperative cognitive function, but only 30 days after surgery (the T4 time point). It failed to demonstrate, however, short-term decrease in length of ICU and hospital stay.

Interestingly, “backward digit span,” “word generation,” and “recall a word list,” whose scores were higher percentage of SG-treated patients (Tables 3, 4), are considered the most difficult tests among those used to evaluate the cognitive domain of PQRS (Kim et al., 2019). Thus, those tests may be the most sensitive for detecting differences in the recovery of cognitive function.

The analysis of the number of correct answers in the “word list” category in the SG group at T3 (Figure 3) revealed an increase relative to their own baseline time (T0), with statistically significant differences (*p* < 0.05), which were maintained 30 days after the surgical procedure (T4). Similarly, a statistically significant difference in the “word generation” category was observed at T4 in the SG group (Figure 4). Therefore, our findings not only indicated quantitative improvement in cognition function (percentage of patients who recovered or equaled the baseline value at different time points) especially at T4, as we shall see below, but they also highlighted significant qualitative improvement in terms of the number of correct answers in the SG group.

On the other hand, both study groups showed significant decrease in the number of responses at 30 min (T1) (Figures 3, 4), a clinically predictable situation after a long and extremely pro-inflammatory procedure such as cardiac surgery. In fact, the accelerated cognitive decline at T1 was predominantly related to impairments in memory and attention/executive function; however, a significant trend toward postoperative cognitive improvement has been detected over time in the SG group. It could be argued that the SG-treated patients perform better in memory, attention, and psychomotor speed, all of them commonly impaired, following cardiac surgery (Glumac et al., 2019).

Among the categories of the physiological domain in the PQRS, recovery of consciousness was also evaluated separately in this study (Table 2). In this sense, the percentage of patients “fully awake” and “obeying verbal commands” 30 min after extubation (T1) was significantly higher in the SG group than in the NG group (85.7 vs. 42.9%, *p* = 0.04). Although the term “fully awake” has seemingly subjective connotations, we tried to obtain data related to orientation, attention, and memory, such as obeying verbal commands and remembering her/his “name, date of birth, and place in which she/he is,” among other questions. Knowing their “name, date and place” (cognitive domain) showed a statistically significant difference (92.9 vs. 57.1%, *p* = 0.049) in the SG group at 30 min (Table 4). All these results are consistent with previous reports (Amorim et al., 2014; Liu and Yin, 2018) and with data obtained from our survey on anesthesiologists. Other studies point out this improvement in terms of “time-to-consciousness” as return of consciousness several minutes faster after reversal with SG compared to NG (Biricik et al., 2019), or “time to recovery” as time to attaining full neuromuscular recovery according to a TOF ratio of > 0.9 from TOF ratio of.25 (Amorim et al., 2014; Claroni et al., 2019).

It has been suggested that muscle stretch receptors can generate signals, which through afferent nerve pathways may cause arousal in the brain. Some authors have put forward the so-called “afferentation” theory of cerebral arousal by proposing that effects on muscle stretch receptors caused by more profound reversal of rocuronium lead to more rapid arousal (Aho et al., 2012). Consequently, the restarting of muscle stretch signal caused by the reversal of NMB decreases the depth of anesthesia and increases electroencephalographic activity and consciousness state, therefore accelerating recovery. In our study, the differences between SG and NG cannot be exclusively explained by faster return of neuromuscular transmission, because both drugs were administered when muscle activity appeared (TOF25) in all the subjects, and the TOF ratio was greater than 90 before returning consciousness in both groups. Therefore, it should be emphasized that both groups achieved the same degree of motor recovery (TOF 90%) before the recovery of consciousness was analyzed. In agreement with other authors, our findings indicate that responsiveness was mainly limited by recovery from the anesthetic drugs rather than by neuromuscular paralysis (Aho et al., 2012; Amorim et al., 2014; Piskin et al., 2016).

On the other hand, it is well-known that the cholinergic system plays a notable role in the decline of cognitive function through nicotinic acetylcholine receptors, which are involved in chemical signaling and regulation of consciousness, memory, and learning (Batistaki et al., 2017; Kim et al., 2019). A central cholinergic deficit caused by disturbances in the cholinergic transmission due to perioperative administration of anticholinergic drugs has been suggested as a possible cause of POCD. This question is especially pertinent for anticholinergic agents that are capable of crossing the blood-brain barrier (BBB) (Batistaki et al., 2017; Kim et al., 2019). SG as a reversal agent for rocuronium lacks anticholinergic activity; similarly, it should be highlighted that NG-treated patients who required anticholinergic treatment during NMB reversal were excluded from the study to avoid confounding and facilitate analysis of both groups.

Our results failed to demonstrate that SG (compared to NG) optimizes the quality of overall postoperative recovery while reducing the length of ICU and hospital stay. In contrast, we found a slight but significant reduction in hospital stay for the NG group (4,2 ± 1,2 vs. 3,14 ± 0,9; *p* = 0.049). We believe that the slightly older age of patients in the SG group (74 ± 3 vs. 70 ± 5; *p* = 0.082) and their worse cardiac operative risk evaluation (logistic EuroScore II) (3,18 ± 1,33 vs. 2,15 ± 0,7; *p* = 0.071) could be factors that, at least clinically, could explain that although the SG patients seem to recover from anesthesia (regain consciousness and have increased alertness) better than the NG ones, their stay in hospital is longer.

Although the global perspective of the patients in terms of “working capacity, daily activities, clarity of thought” and “satisfaction with the anesthetic care” analyzed at T4 all showed better scores in the SG group than in the NG group, the differences found were not statistically significant. However, the pain domain at T4, as has been already pointed out, was significantly better valued in the SG group, without being able to infer a causal relationship with SG, when both groups received a similar multimodal analgesic strategy. Certainly, there is evidence that patients who undergo surgeries, such as coronary artery bypass graft and joint replacement surgery, have decreased pain and inflammation and benefited from improved quality of life and, perhaps, persistent cognitive improvement (Needham et al., 2017). Furthermore, some prospective studies have shown that when surgery treats pain successfully, cognitive function improves (Needham et al., 2017). Undoubtedly, our current data are inadequate to verify or refute a hypothetical relationship between the significant reduction in pain and supposed postoperative cognitive improvement (POCI) observed at 30 days (T4) in SG versus NG, but it should be noted that establishing such a relationship was not one of the objectives of this study.

In addition to the clinical data discussed, we have tried to explore the mechanisms of action potentially involved in this relatively novel molecule such as SG, which could justify this apparent and presumed relationship with POCI. Experimental data show that surgery induces the release of proinflammatory cytokines in the brain, including the hippocampus, disrupting memory and learning functions (Needham et al., 2017; Klinger et al., 2018). Inflammation generates lethargy, anorexia, fever, and cognitive dysfunction, all of which promote rest and allow wound healing to proceed (Needham et al., 2017). The marked systemic inflammation in the immediate postoperative period probably contributes to postoperative delirium or early POCD but may produce a longer-term injury in certain vulnerable patients (Needham et al., 2017).

Currently, a very decisive hypothesis of POCD development includes systemic inflammatory response syndrome (SIRS) induced by cardiac surgery and CPB itself (Bhamidipati et al., 2017; Needham et al., 2017; Glumac et al., 2019). SIRS contributes to BBB leakage as well as development of cerebral edema and cerebral inflammation (Glumac et al., 2019), and has possibly a crucial role in POCD pathogenesis (Bhamidipati et al., 2017; Glumac et al., 2019). Lesions underlying POCD probably affect the hippocampus, which is closely related to memory (Glumac et al., 2019) and pronouncedly sensitive to hypoxic injury. A number of different and interesting interventions have been described in the literature on cognitive functions, such as effects of valerian root, minocycline, statins, lidocaine, ketamine, and noble gas xenon among others, predominantly through their anti-inflammatory activity (Glumac et al., 2019).

### Experimental Study

The experimental study was designed to explore cognitive effects after mild surgery in experimental groups treated with or without SG. Our results indicate that immediately after surgery, during the recovery period (1 h), there was not any difference in the motor activity between the experimental surgery groups. Even not comparing with other similar clinical drugs, these results are different from those expected by the clinical observation, where it faster recovery of patients treated with SG has been reported, as was reflected on the survey on anesthetists and substantiated by previous publications (Biricik et al., 2019). This difference could be explained by three reasons: the type of surgery, types of parameters used to analyze recovery, and species differences. Our animal model of surgery was less invasive and of much lower severity grade compared to the clinical cases of reference. Besides, data obtained in the clinical setting were not substantially based on motor parameters, and, finally, there is an evident physiological difference between rats and humans that could influence their capacity for recovery differently (Ellenbroek and Youn, 2016).

Following the motor activity assessment, cognitive recovery was analyzed by a spatial behavior test. All the groups were trained before surgery, and the learning index was the result of the difference between the behavior results prior to and after surgery. Our results indicate that cognitive impairment caused by surgery was reversed by the treatment with SG. This may suggest that a mild surgery can induce mild cognitive deficits and, second, that this cognitive deficit can be at least partially reduced by SG administration. As far as we know, this is the first experimental data reporting the effect of SG on post-surgery cognitive recovery, but our results do not rule out that other muscular antagonists can mimic the beneficial effects reported here.

To assess the surgery and effects of SG on spatial memory, the expression of neuroinflammation markers in the rat hippocampus was analyzed. The level of inflammation of the hippocampus has been related to spatial memory capacity (Hernandez-Rabaza et al., 2015) and hippocampus-dependent POCD.

ATP is a neuromodulator that has been proposed as a signal candidate between the peripheral and central nervous systems. It has been postulated as a possible inducer of inflammation. The statistical analysis of the purinergic P2 × 7 receptor expression data (from the western blot assay) did not show significant differences among the groups but revealed tendency to high values in the surgery groups. Further analyses are required to elucidate the initial modulation role of P2 × 7 in inflammation. Similarly, the analysis of classic cellular pro-inflammatory markers indicated that there are no significant differences in the astrocyte (GFAP) and microglia-macrophage (Iba1, and CD68) markers. Additionally, immunohistochemistry analysis of the astrocytes showed no differences in the hippocampus expression of the astrocyte marker. However, the immunohistochemistry analysis indicated an alteration of the microglia expression and activation patterns by the surgery, which was reduced by SG. Differences in the results between Western blot and immunohistochemistry techniques can be explained by the sensitivity of both techniques, and further investigation is needed to confirm our observations.

The possible alteration in microglia activation may be reflected in the polarization of the microglia, and in this line, significant differences were seen in microglia M2 marker expression: the surgery group treated with SG showed higher IL4 and YM-1 protein expression levels. M2 microglia has been related to decrease in inflammation process (Chhor et al., 2013). The microglia activation data, based on morphological results, support the anti-inflammatory effect and suggest a possible new role for SG, opening a connection between SG and POCI through the regulation of neuroinflammation.

The reduction of neuroinflammation with SG could be related to a potential neuroprotection role. Recently, the neuroprotective potential of SG in an animal model of head trauma has been pointed out (Mucuoglu et al., 2021). Nevertheless, before confirming the possible neuroprotective or anti-inflammatory role of SG, many questions must be unveiled. SG cannot cross the BBB because of its high molecular weight; hence, it is difficult to understand how it can modulate neuroinflammation. However, neuroinflammation is regulated by peripheral signals through several molecular pathways, and SG may influence them. In fact, it has been reported that under surgical procedures, intracellular mediators such as RNA released from damaged tissue initiate an immune-mediated response that triggers the central immune response that amplify neuroinflammation through the vagus afferent nerve or partially permeable BBB, which has been described in aging, certain neurological pathologies (Noll et al., 2017; Lin et al., 2020), and exposure to volatile anesthetics such as sevoflurane (Satomoto et al., 2016). Moreover, immune stimulation of the central nervous system by surgery induces glial cell activation, prompting the release of inflammatory cytokines. This fact has been linked to increase in cognitive disorders particularly described in elderly patients undergoing surgery (Kang et al.; Liu and Yin, 2018). In this sense, the state of microglia activation determines the grade of neuroinflammation and emerges as an underlying mechanism for POCD. Furthermore, as stated earlier, peripheral parameters affect the regulation of neuroinflammation. SG has been reported to affect peripheral parameters, such as coagulation reduction (Kang J. H. et al., 2020; Kang W. S. et al., 2020), return of neuromuscular signals (Biricik et al., 2019), and peripheral inflammation (Yeşiltaş et al., 2021). Moreover, plasma inflammatory and neuronal injury markers have been reported by surgery and anesthesia procedures (Subramaniyan and Terrando, 2019).

### Limitations of the Study

Certainly, the study has some important limitations. The clinical one uses a very small sample size, probably caused by an excessive effort to reduce selection and procedural bias. We have not explored higher doses of SG within the authorized dose range, and we have not performed a selection of specific neurocognitive tests. Both the experimental and clinical studies could have been carried out for longer periods.

The experimental animals were sacrificed in a short time post-surgery to analyze certain biological parameters during the awareness recovery period because that was the period for which physicians reported an evident effect by SG. This approach conditioned other parameters, especially the selection of animal model, which was conditioned by the behavior trial. As a result, carrying out a cardiopulmonary bypass in the rats was avoided because of its length and severity, both of which would affect post-surgical behaviors test performance, mainly compromising the swimming capacity of the animals. Laparotomy is a milder surgery; therefore, its effects cannot be correlated with the clinical data from severe cardiac surgery in humans. For example, the cognitive alteration reported in the animal model was mild spatial memory impairment instead of loss of memory or cognitive incapacity to resolve the task. It is remarkable, nevertheless, that a mild surgery induces mild memory impairment that was reverted by treatment with SG.

Additionally, the behavior task conditioned the brain area selected. The hippocampus was the brain area selected for the analysis because of its strong relation with spatial memory and the Morris Water Maze task. However, other brain areas can be affected and considered for future analysis. Moreover, other cellular processes, such as neurodegeneration and cellular oxidative stress, and even the permeability of the BBB in addition to inflammation, may be affected by surgery and certain pharmacological treatments. These variables should be explored in future research to unveil the possible cognitive role of SG. Finally, both further histological and molecular analyses should be performed to consolidate or refute the microglia activation and polarization effects of SG.

Considering the conditions and limitations of the clinical and experimental approaches, conclusions on the effect of SG should be cautious. Future work should avoid the limitations of this study and engage in finding reliable inflammatory and/or biochemical markers that could allow for a strong and reliable correlation of the experimental findings with clinical data.

## Conclusion

To our knowledge, this study is the first that attempts to demonstrate the therapeutic effect of SG on postoperative functional and cognitive recovery in patients undergoing aortic valve replacement by cardiopulmonary bypass and the ERACS approach with the postoperative quality and functional recovery scale (PQRS). The clinical study has been complemented with an experimental study that points out a possible mechanism of action for SG based on its potential anti-inflammatory role on the hippocampus.

## Data Availability Statement

The raw data supporting the conclusions of this article will be made available by the authors, without undue reservation.

## Ethics Statement

The studies involving human participants were reviewed and approved by the Ethics Committee, from the Health Department of the Hospital Ribera, Valencia, Spain. The patients/participants provided their written informed consent to participate in this study. The animal study was reviewed and approved by 2018/VSC/PEA/0081.

## Author Contributions

VM performed conceptualization. VM and VH-R designed methodology. ML, AA, and CP performed Investigation. VR performed formal analysis. VM and VH-R writing and preparation of the original draft. VR, VM, and VH-R performed writing, review, and editing. All authors substantially and directly contributed to the study and read and agreed to the published version of the manuscript.

## Conflict of Interest

The authors declare that the research was conducted in the absence of any commercial or financial relationships that could be construed as a potential conflict of interest.

## Publisher’s Note

All claims expressed in this article are solely those of the authors and do not necessarily represent those of their affiliated organizations, or those of the publisher, the editors and the reviewers. Any product that may be evaluated in this article, or claim that may be made by its manufacturer, is not guaranteed or endorsed by the publisher.
