# Supplementary material for: Potential Neuroprotective Role of Sugammadex: A Clinical Study on Cognitive Function Assessment in an Enhanced Recovery After Cardiac Surgery Approach and an Experimental Study
Source: Front Cell Neurosci. 2022 Feb 21;16:789796. doi: 10.3389/fncel.2022.789796 (PMC8900639; doi:10.3389/fncel.2022.789796)
Supplement: Supplementary file 1 [file Presentation_1.pdf]

Supplementary file. Appendix section

Appendix A:

Sugammadex: current use of the treatment

Expert opinion

*Mandatory mark one option

1. What work experience do you have, including your training as a resident? *

Mark only one option

- 4 years
- 5-10 years
- 11-15 years
- 16-20 years
- 20 years

2. As an anesthesiologist, and within the surgical area, what specialty usually occupies most of your working time?

Mark only one option

- General Surgery
- Cardiothoracic
- Traumatology
- ORL
- Other:

3. During a Rapid Sequence Induction (RSI) scenario, which neuromuscular relaxant drug do you usually use? *

Mark only one option

- Suxamethonium / Succinylcholine
- Rocuronium
- None
- Other:

4. Monitors (e.g., TOF) muscle relaxation *

Mark only one option

Never 1 / 2 / 3 / 4 / 5 / Always

5. Uses rocuronium (or vecuronium) to induce and/or maintain general anesthesia *

Mark only one option

Never 1 / 2 / 3 / 4 / 5 / Always

6. Regarding the reversing pharmacological agents (anticholinesterase/Sugammadex) of neuromuscular blockade. You use Sugammadex when you have used rocuronium (or vecuronium) as blocking agents *

Mark only one option

Never 1 / 2 / 3 / 4 / 5 / Always

7. Sugammadex helps accelerate recovery of consciousness/alertness, regardless of its contribution to restoring the motor ability

Mark only one option

Totally disagree 1 / 2 / 3 / 4 / 5 / Totally agree

8. Sugammadex can help to optimize the subjective feeling of well-being of patients after anesthesia awakens *

Mark only one option

Totally disagree 1 / 2 / 3 / 4 / 5 / Totally agree

9. Sugammadex can be an essential tool in the new Accelerated Recovery (fast-tracks) or Enhanced Recovery After Surgery (ERAS) programs *

Mark only one option

Totally disagree 1 / 2 / 3 / 4 / 5 / Totally agree

10. In your department, is Sugammadex readily available in your unit?

Mark only one option

- Yes
- No
- Sometimes
- DK / Na

11. Indicate the side effects that you have supposed or related to the use of sugammadex (indicate all observed) *

Mark only one option

- Anaphylaxis
- Bronchospasm
- Headaches
- Dysgeusia
- Nausea and vomiting
- Weakness
- Urticaria
- Abdominal pain
- I have not observed any
- Other:
