# Supplementary material for: Potential Neuroprotective Role of Sugammadex: A Clinical Study on Cognitive Function Assessment in an Enhanced Recovery After Cardiac Surgery Approach and an Experimental Study
Source: Front Cell Neurosci. 2022 Feb 21;16:789796. doi: 10.3389/fncel.2022.789796 (PMC8900639; doi:10.3389/fncel.2022.789796)

Additional supplementary data

Index

1. **Immunohistochemistry supplementary data**
2. **Behavior supplementary data**
    - **Morris Water Maze, training sessions (before surgery or treatment applications).**
    - **Morris Water Maze, average swimming speed, test session (after surgery and treatment applications).**

1. Immunohistochemistry supplementary data

Iba1 analysis expression in the hippocampus. Three different areas were analyzed in the hippocampus, the mean values are represented in the graph. Areas: dentate gyrus, molecular layer, and CA1. An image of the CA1 is added as an example of the staining.

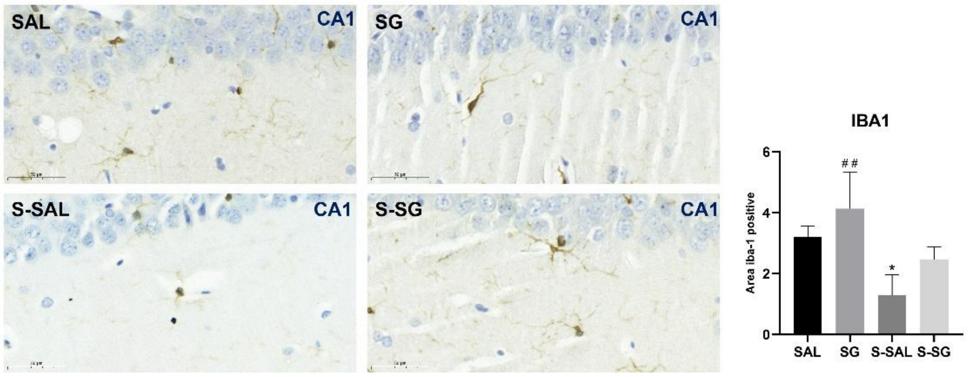

Representative images of the microglia marker Iba1, brown mark, in the region proximal to the CA1 region, and a graph from the results. Images and graph, SAL and SG, no surgery treated with saline or sugammadex, respectively, and S-SAL and S-SG, groups with surgery treated with saline or sugammadex, respectively. Graph, * significant different with SAL (p=0,049), and # # significant different with SG (p=0,0064).

GFAP analysis in the hippocampus. Three different areas were analyzed with no differences detected, as can be appreciated in the graphs. Areas: dentate gyrus, molecular layer, and CA1. An image of the CA1 is added as an example of the staining.

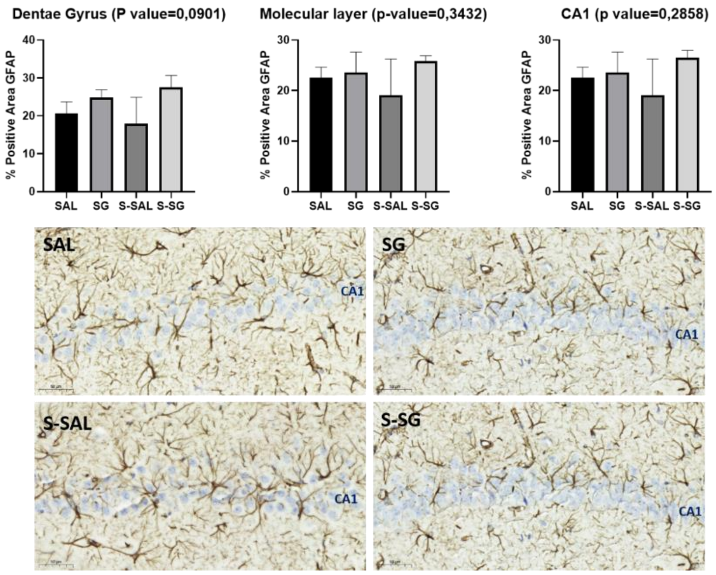

Representative graphs of the mean values of GFAP in the three mentioned hippocampus regions. Below, representative images of the astrocyte marker GFAP, brown mark, in the region proximal to the CA1 region, and a graph from the results. Images and graph, SAL and SG, no surgery treated with saline or sugammadex, respectively, and S-SAL and S-SG, groups with surgery treated with saline or sugammadex, respectively.

2. Behavior supplementary data

Pre-surgery training in the Morris Water Maze. Percentage in the platform quadrant. Groups mean values during three pre-training days. No significant differences between the experimental groups were detected, but a strongly significant difference between day 3 and days 1 and 2, indicating learning of the task. Highlight the values during the first and second day around 25%, as is expected by chance. Remind that during this period, no surgery or treatment was applied, the group assignation was referred to the posterior day 4.

Statistical information. Two-way RM ANOVA (Graph Pad Prism 4 software; GraphPad Software Inc. San Diego, CA).

Row Factor (Experimental Group)	p- value: 0,4874
Column Factor (Time)	p-value: 0,0002**

Tukey's multiple comparisons test	Adjusted P Value
DAY 1 vs. DAY 2	0,9165
DAY 1 vs. DAY 3	0,0002
DAY 2 vs. DAY 3	0,0013

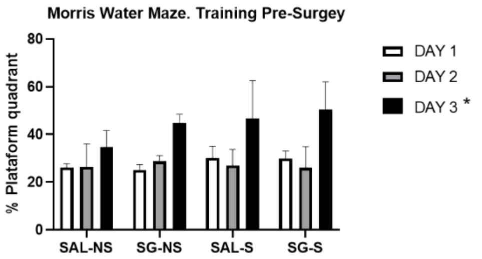

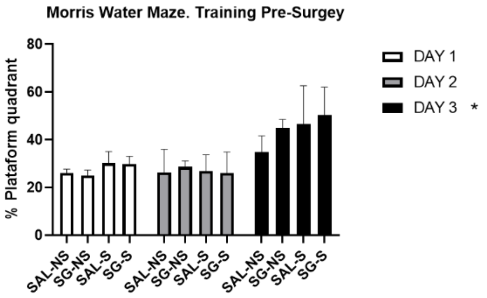

Experimental groups assignation. Results previous surgery or treatment application

Morris Water Maze. Average swimming speed, **in day fourth, post-surgery.** Table with the mean values and standard error and representative graph. The units are arbitrary units calculated by the specific software (ViewPoint. Behavior Technology). No differences were shown between groups, as revealed by the statistical comparison (ANOVA, p-value: 0.2862).

	SAL-NS	SG	S-SAL	S-SG
Mean	23,7	21,53	24,22	23,14
Std. Error of Mean	0,3958	0,6225	1,514	1,624

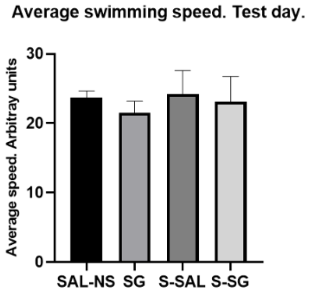

Supplement: Supplementary file 2 [file Data_Sheet_1.pdf]
